# Association between the built environment and moderate to vigorous leisure-time physical activity among suzhou adolescents: a cross-sectional study

**DOI:** 10.1186/s12889-023-16243-0

**Published:** 2023-07-10

**Authors:** Hewu Lv, Rui Wang

**Affiliations:** 1 College of Sports Science, Nantong University, Nantong, 226019 China; 2 Student Affairs Office, Nantong University, Nantong, 226019 China

**Keywords:** Leisure-time physical activity, MVPA, Health, Built environment, Adolescent

## Abstract

**Introduction:**

Cardiovascular disease and obesity are both significantly influenced by physical inactivity. A rapidly expanding corpus of research contends that features of the built environment might encourage adolescents to lead active lives. There are still issues with the present evidence for determining which aspects of the built environment give adolescents the opportunity to engage in leisure-time physical activity (LTPA). This study looked at the relationship between the characteristics of the built environment and moderate-to-vigorous leisure-time physical activity (Leisure-time MVPA) of adolescents.

**Methods:**

2628 adolescents between the ages of 11 and 18 were chosen as study participants from 19 Suzhou urban communities. They must have resided in the neighborhood for longer than six months and be permanent residents there. The International Physical Activities Questionnaire (*n* = 2628) and the Neighborhood Environment Walkability Scale for Chinese Children (NEWS-CC) were used to collect the data. LTPA are connected to different modes: Walking, leisure-time MPA, and leisure-time VPA. Univariate analysis and multinomial logistic regression were used to screen for potential associations between the built environment and the leisure-time MVPA in adolescents.

**Results:**

Univariate analysis of the general demographic and built environment showed statistically significant differences in gender, residential density, accessibility, pedestrian safety, aesthetic and security (*P* < 0.05). Step by walking reference category, security (*P* < 0.05, OR = 1.131) were associated with adolescents' leisure-time MPA, aesthetics (*P* < 0.05, OR = 1.187) were associated with adolescents' leisure-time VPA, they both have a significant positive correlation.

**Conclusion:**

Security was positively associated with adolescents' leisure-time MPA, aesthetics was positively associated with adolescents' leisure-time VPA. This suggests that built environment may associated with leisure-time MVPA of Suzhou adolescents.

## Background

Regular participation in leisure-time physical activity (LTPA) is essential for the health and well-being of adolescents [1]. Adolescent health can be improved by increasing daily LTPA. LTPA is widely recognized for its ability to prevent and treat a wide range of physical and psychological disorders. Regular LTPA during childhood is believed to lower the incidence of serious health problems [2]. Regular participation in a variety of organized LTPAs is associated with improved personal health and well-being, including better cardiovascular health, mental health, life satisfaction, quality of life [3], reduced stress [4], lower obesity levels [5], and improved academic adjustment [6]. Current estimates show that only 40% to 50% of adolescents aged 6 to 11 and 6% to 11% of adolescents aged 12 to 15 participated in at least 5 of the previous 7days of 60min of moderate-intensity activity in the US [7].

LTPA has become particularly important for Chinese children to increase their overall physical activity level because they don't perform household chores [8]. Based on the SLOTH (sleep, leisure time, occupation, transportation, and home-based activities) model, which outlines the domains of physical activity, LTPA in adolescents usually occurs in the built environment of the community [9], and the built environment is one of the key settings in which adolescents can freely engage in LTPA [10]. The built environment of a community is a crucial setting for promoting children's LTPA and health. A growing number of studies have linked adolescents’ LTPA to features of the built environment in their locked-in neighborhoods [11]. Zou systematically reviewed the relationship between residential density and childhood obesity and found that residential density may be associated with childhood obesity [12]. Girls typically accumulate less MVPA and more sedentary time than boys, and these findings are supported by the literature. The literature further points out that built environments may give boys privileges because they have equipment that they are interested in, and these built environments do not support girls' interests [13]. The same may be true of research findings in the field of LTPA. With this in mind, it seems necessary to provide girls with space and equipment, as well as a suitable built environment, to stimulate their LTPA. Therefore, hypothesis H1: Adolescents' leisure-time MVPA differs by gender, with boys being significantly higher than girls.

Little is known about the specific associations between built environment characteristics (e.g., residential density, diversity) and adolescents' leisure-time MVPA. Sallis and colleagues paid close attention to the associations between the built environment and physical activity [14]. The street connectivity, walkability, mixed land use, and residential density of the built environment received increasing focus, and a 5% increase in walkability was found to be associated with a per capita 32.1%increase in time spent in physically active travel [15]. Another study by Cervero showed that higher residential density was associated with a lower share of car commuting and that residential density had a more significant effect on commuting mode choice relative to mixed land use [16]. Residential density is one of the important built environment factors that affect leisure-time MVPA among adolescents. It can positively affect LTPA among adolescents. According to a recent study, individuals are more likely to walk and bike in areas with higher residential densities, a variety of land uses (for example, stores nearby residences), and linked streets. Some studies have shown that higher residential density is associated with a decrease in time spent participating in physical activity and an increase in sedentary time and is positively correlated with leisure-time MVPA in adolescents [17]. When compared to less populous areas, densely populated areas are associated with higher levels of physical activity and outdoor exercise [18, 19]. Some studies have also concluded that there is no correlation between residential density and leisure-time MVPA [20]. This suggests that the results of studies on the relationship between residential density and leisure-time MVPA are inconsistent, mainly due to the large variability in the samples selected, between cities and regions and the different methods of measuring the built environment, and that research in this area needs to be further developed.

The availability and accessibility of necessary infrastructure for walking and cycling were contentious. Evidence suggests that large-scale infrastructure and built-environment initiatives to promote cycling are likely to be necessary, emphasizing the importance of creating safe, designated (or segregated), connected, and supportive routes and urban environments [21]. Adolescents living in "traditional" or "walkable" neighborhoods engage in more physical activity per week than those living in "suburban" neighborhoods. Most of the existing studies have proven that the built environment plays a significant role in adolescent physical activity and health. When adolescents perceive that the neighborhood is better landscaped, their LTPA such as walking, increases significantly, and they are more likely to achieve their recommended amount of physical activity [22]. Urban planners have proposed ideas such as installing lighting systems in the built environment or improving aesthetics to encourage or support LTPA for adolescents [23]. Therefore, the built environment characteristics of communities must be known by stakeholders that have an impact on adolescents’ LTPA [24] and improve their well-being [25]. Although the relationship between the built environment and adolescents’ LTPA has been established, leisure-time MVPA and built environment outcomes at determined times have not received as much attention. Limited research has addressed whether these built environment characteristics act as potential facilitators for participation in leisure-time MVPA. Hypothesis H2: Residential density, diversity, accessibility, pedestrian safety, aesthetics, and security positively influence adolescents' leisure-time MVPA. Traffic hazards, and obstructions that negatively influence adolescents' leisure-time MVPA. The main aim of this study was to examine the associations between the built environment and adolescents' leisure-time MVPA as assessed by questionnaires.

Given the above research deficiencies, this study evaluates the relationship between the built environment and leisure-time MVPA of adolescents. The International Physical Activity Questionnaire (IPAQ) and the Environment Walkability Scale for Chinese Children (NEWS-CC) were used to analyze in detail the effects of built environment such as residential density, diversity, security, aesthetics and other factors on leisure-time MVPA of adolescents in Suzhou, so as to determine the quantitative relationship between the built environment and leisure-time MVPA of adolescents.

## Methods

### Sampling

**Fig. 1:**
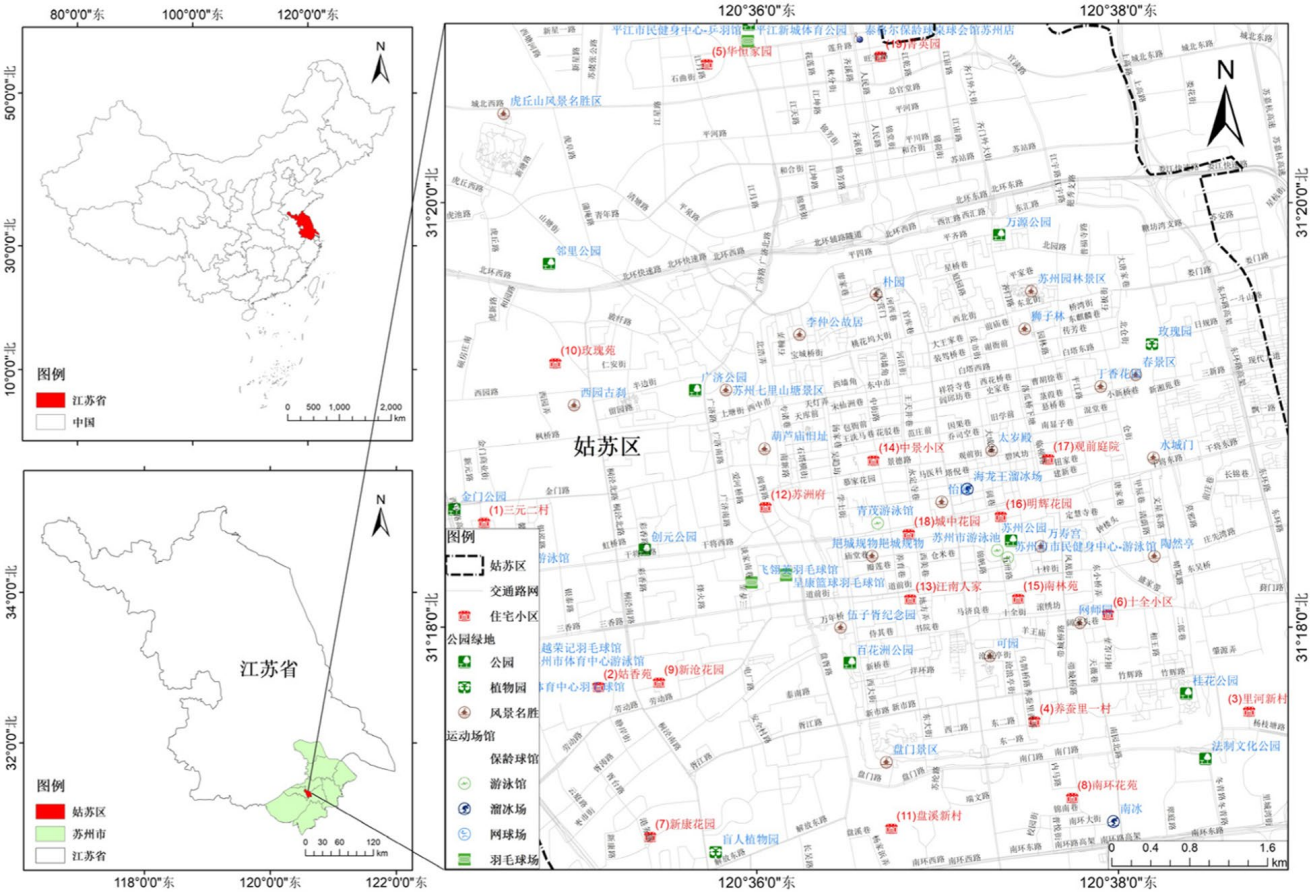
The distribution of selected communities in Gusu District

This research adopted a cross-sectional research design, the key element of which was the LTPA of adolescents in the built environment of a community. Using a simple random sampling method, 19 communities were selected in Gusu District, Suzhou City (Fig. 1). The built environment in Gusu District of Suzhou has a dense road network, and there are abundant trails or alleys that are only suitable for walking and cycling, high connectivity, and high walkability. Sports service facilities are numerous and evenly distributed. Gusu District of Suzhou has smart growth characteristics. Smart growth is an overall approach to development that encourages a mix of building types and uses, diverse housing and transportation options, development within existing neighborhoods, and robust community engagement. The 10 principles, such as mixing land uses, creating walkable neighborhoods, preserving open space, farmland, natural beauty, and critical environmental areas, etc.,conducive to the promotion of LTPA for adolescents, so adolescents are more engaged in leisure-time MVPA in this environment.

In order to be able to collect high quality data within a week, we employed trained questionnaire interviewers and utilized technology, making it possible to efficiently collect a large number of samples within a short period of time. We employed eight experienced and skilled questionnaire interviewers to perform sample collection, and these eight long-term trained questionnaire interviewers can greatly increase the efficiency of the process. 8 questionnaire interviewers reached the communities to communicate with adolescents and parents between April 13 and 19, 2019. We also utilized the largest reproducible questionnaire platform in China, named"Questionnaire Star", and the questionnaire platform could be utilized to facilitate the collection of a large number of samples in a short period of time. In order to prevent selection bias, and after clarifying the study topic, adolescents and their parents were invited to complete an informed consent form and an online questionnaire. We have adopted on-site interviews and manual screening methods. The IP address was set to allow filling only once. Inclusion criteria: (i) those who had lived in the community for at least 6 months; (ii) those aged 11 to 18 years; (iii) those who were willing to participate in the study and had signed an informed consent form by themselves and their guardians. Exclusion criteria: (i) those with chronic diseases taking medication and psychiatric disorders (ii) those who could not complete normal physical activities; (iii) those who did not want to sign the informed consent form. In addition, questionnaires that took too long(> 30 min) or too short(< 2 min) to answer and those that showed significant outliers were screened by manual judgment. A total sample of 3112 respondents was collected, 484 invalid questionnaires were deleted(Less than 6 months *n* = 21, Less than 11 years *n* = 37, Greater than 18 years *n* = 45, Did not sign informed consent *n* = 26, Could not complete physical activities *n* = 44, Significant outliers *n* = 311), 2,628 valid questionnaires were finally obtained. Boys and girls, respectively, make up 1364 (51.9% of the sample) and 1264 (48.1%). This research protocol was approved by the Ethics Committee of Nantong University, and participants involved in this study have given written informed consent under the Declaration of Helsinki.

### Variables

This study evaluates the relationship between the built environment and leisure-time MVPA of adolescents based on the IPAQ and NEWS-CC. The spatial cognition survey data from this study will establish the quantitative relationship between the built environment and adolescents' leisure-time MVPA. Leisure-time physical activity levels were obtained using the IPAQ, which is currently recognized as a valid and widely used tool for measuring physical activity levels and includes work-related, transportation-related, domestic gardening-related, and leisure-time physical activities. As seen in Table 1,The level of leisure-time physical activity in the IPAQ consisted of three components: walking physical activity, moderate leisure-time physical activity, and vigorous leisure-time physical activity. Weekly vigorous leisure-time physical activity levels (MET-min/w; metabolic equivalent of energy) were calculated according to the IPAQ working group recommendations. Adolescents' leisure-time physical activities were categorized as walking, leisure-time MPA, and leisure-time VPA.

**Table 1:** Explanatory information on independent variables

Criteria		Standards
IPAQ	Leisure-time VPA	1. The total of vigorous physical activities is ≥ 3day, and the total physical activity level per week is ≥ 1500 MET-min / W
		2. The total physical activity of the three intensities is ≥ 7days, and the total physical activity level per week is ≥ 3000 MET-min / W
	Leisure-time MPA	1. Meet all kinds of vigorous physical activities for at least 20min every day, totaling ≥ 3days
		2. Meet all kinds of moderate intensity and / or walking physical activities for at least 30min every day, with a total of ≥ 5days
		3. The total physical activity of the three intensities is ≥ 5days, and the total physical activity level per week is ≥ 600 MET-min / W
	Leisure-time LPA	1. No physical activity was reported
		2. Some physical activities have been reported, but they do not yet meet the above criteria for moderate and vigorous grouping
NEWS-CC	Residential density	Number of households per residential area
	Diversity	Percentage of total parcel area for different land-uses (e.g., commercial, industrial, recreational, residential uses, etc.)
	Accessibility	Distance (network and/or straight-line) to the nearest specified destinations (e.g., parks, recreation facilities, and playgrounds)
	Pedestrian safety	Sidewalk coverage measured by sidewalk length divided by road length
	Aesthetics	Greenness/vegetation measured by normalized difference vegetation index
	Traffic hazards	Street width (excluding sidewalk), likely to affect the volume of traffic and incidents of accidents
	Security	Number of crimes per 100,000 people (includes violent, property crimes)
	Obstruction	Dead ends, ramps, railway, viaduct

*IPAQ* International physical activities Questionnaire, *NEWS-CC* Neighborhood Environment Walkability Scale for Chinese Children, *Leisure-time LPA* Low Leisure-time physical activities, *Leisure-time MPA* Moderate Leisure-time physical activities, *Leisure-time VPA* Vigorous Leisure-time physical activities

As seen in Table 2. the questions in the IPAQ questionnaire involve” During the past 7days, on how many days were you physically active for a total of at least 60min?”, and”In the past 7days, have you participated in physical exercises with heavy physical activities (such as aerobics, running, fast cycling, swimming, football, and basketball activities, etc.,) with a duration exceeding 10min per day?”. As adolescents are less likely to be involved in work and domestic gardening-related physical activities, only the leisure-time physical activities section of the questionnaire was administered to adolescents in this study.

**Table 2:** Questionnaire sample

Criteria	IPAQ Question items
IPAQ	In the past 7days, how many days have you gone for a walk that lasted more than 10min? (not including the walking time you have described) days/weeks
	How much time do you spend walking every day?
	In the past 7days, have you participated in vigorous physical activity (such as aerobics, running, fast cycling, swimming, football and basketball activities, etc.) for more than a few days and 10min? (Not including the walking time you have described.)
	How much time do you spend on vigorous physical activity every day?
	In the past 7days, have you participated in moderate physical activity (such as fast walking, ballroom dancing, bowling, table tennis, badminton, etc.) for more than a few days and 10min?
	How much time do you spend on moderate physical activity every day?
	NEWS-CC Question items
Residential density (6)	Are there any one-family residential homes,1–3, 4–6, 7–12, 13–20, or more than 20-story residential homes (e.g., villas, village houses, tenement houses, public rental housing, private estates) in your neighborhood?
Diversity (23)	How long would it take you to walk from your house to the nearest leisure facilities/shops/cycling/hiking or walking trail, or other places?
Accessibility (5)	The shop is within walking distance
	There are many places within walking distance of my home
	It's easy to walk from my home to the bus station (bus, subway or train)
	The distance between junctions near my house is usually shorter
	There are many different routes to get from one place to another in my neighborhood
Pedestrian safety(6)	Most streets in my neighborhood have sidewalks
	The road and sidewalk near my house were separated by parked cars
	There is grass or vegetation between the street and the sidewalk near my house
	My neighborhood is well lit at night
	From your own home, you can easily watch pedestrians and cyclists on nearby streets
	In the street near my home, there are zebra crossings and traffic lights to help pedestrians cross the busy road
Aesthetics(4)	There are trees along the street near my house
	There are many interesting things to see on a walk in my neighborhood
	There are many fascinating natural sights near my home
	The buildings near my home are attractive
Traffic hazards(3)	The street near my house is very heavy with traffic, so it is difficult or unpleasant to take a walk
	Traffic on most nearby streets is usually slow
	Most drivers in my neighborhood exceed the posted speed limit
Security(3)	You think your neighborhood is safe
	You think your neighborhood is very safe during the day, which makes you feel safe to go out and walk
	You think your neighborhood is very safe at night, which makes you feel safe to go out and walk
Obstruction(3)	There are not many dead ends in the streets near my house
	The streets near my house are hard to walk on
	There are so many obstacles to walking around my neighborhood that it is difficult to get from one place to another (e.g.,highways, railways, rivers, canyons and hillsides)

The NEWS-CC [26]was used to collect data on the built environment. As seen in Table 2. the questions in the NEWS-CC questionnaire involve “How long would it take you to walk from your house to the nearest shops or other places?”,” Leisure facilities and places in your neighborhood”,” Types of residential homes in your neighborhood”,etc. Residential density(Number of households per residential area), land use diversity (Percentage of total parcel area for different land-uses, e.g., commercial, industrial, recreational, residential uses, etc.), land use mix accessibility (Distance network and/or straight-line to the nearest specified destinations e.g., parks, recreation facilities, and playgrounds), pedestrian safety(Sidewalk coverage measured by sidewalk length divided by road length), aesthetics(Greenness/vegetation measured by normalized difference vegetation index), traffic hazards(Street width excluding sidewalk, likely to affect the volume of traffic and incidents of accidents), security (Number of crimes per 100,000 people includes violent, property crimes), and obstruction (Dead ends, ramps, railway, viaduct) were some of the neighborhood features. In use in several nations, the scale has been shown to be valid and reliable [27, 28]. The items were assessed on a four-point Likert scale (1 = strongly disagree; 4 = strongly agree), with the exception of land use mix-diversity and residential density. Land use mix-diversity items inquired about the walking distance from children's homes to a list of 20 places; responses ranged from > 30-min walking (coded as 1) to 1- to 5-min walking (coded as 2). (coded as 5). Residential density questions inquired about the frequency of six different types of houses, ranging from single-family homes to apartments with 20 floors or more; responses ranged from none (marked as 1) to all (coded as 5). Cerin and colleagues then estimated the residential density subscale score using the procedure [29]. The scale's Chinese translation has undergone several revisions in China. Using the NEWS-CC updated by Professor He Gang as the basis for this study, the findings showed that the NEWS-CC had satisfactory test–retest reliability, with subscale ICCs ranging from 0.47 to 0.86. Residential density and mixed land use diversity are two portions of the scale that are based on objective built environment surveys and were not subjected to any reliability testing. A subjective built environment survey. The subjective built environment survey includes: accessibility (Cronbach's alpha = 0.827), pedestrian safety (Cronbach's alpha = 0.814), aesthetics (Cronbach's alpha = 0.882), traffic hazards (Cronbach's alpha = 0.779), security (Cronbach's alpha = 0.911), and obstruction (Cronbach's alpha = 0.765), indicating good internal consistency and high reliability of this scale.

### Statistical analysis

SPSS 22.0 software was used for the analysis in this article. A total sample of 3112 respondents was collected, 484 missing data were deleted, and 2,628 valid questionnaires were finally obtained. The leisure-time physical activities identified walking, leisure-time MPA, and leisure-time VPA groups. Logistic regressions were performed in the crude and adjusted analyses. In the model, the covariates were gender. The Kolmogorov–Smirnov test found that the walking (*p* < 0.05), and leisure-time MPA (*p* < 0.05), leisure-time VPA (*p* < 0.05), LTPA (*p* < 0.05) of boys and girls all showed a non-normal distribution. A descriptive analysis was performed using the median and the interquartile range. The Mann–Whitney’s U-test (continuous variables) was employed to explore the dependence between gender and LTPA. The demographic variables were reported as percentages, and the built environment was reported as the median and the interquartile range. The chi-square test was used to determine statistical significance. The method of multinomial logistic regression is used to test the model and explore the impact of factors in the built environment on adolescents' leisure-time MVPA. Beta coefficient represents the change in log odds of being in a particular category of the dependent variable associated with a one-unit increase in the corresponding independent variable, it shows how much the odds of being in a certain category change with a one-unit increase in an independent variable relative to a reference category.

## Results

### Descriptive analysis

The gender characteristics of adolescents reflect individual demographics and are important factors associated with individual leisure-time MVPA. As seen in Table 3, there are significant differences in walking (Z = -1.999, *P* < 0.05), leisure-time MPA(Z = -4.999, *P* < 0.05), leisure-time VPA (Z = -2.814, *P* < 0.05), LTPA (Z = -5.207, *P* < 0.05) and between boys and girls.

**Table 3:** Results of the Mann–Whitney U test considering LTPA by gender

Dimension	Gender	Minimum	Maximum	Median (IQR)	Z	*P*
Walking	Boy	0	4389	185(0–346.5)	-1.999	0.046^*^
	Girl	0	3696	99(0–330)		
leisure-time MPA	Boy	0	5040	240(0–480)	-4.199	0.000^***^
	Girl	0	5040	160(0–400)		
leisure-time VPA	Boy	0	10,080	400(0–960)	-2.814	0.005^**^
	Girl	0	10,080	320(0–800)		
LTPA	Boy	0	14,120	932(400–1809.5)	-5.207	0.000^***^
	Girl	0	15,509	725(323–1492)		

^***^ indicates *P* < 0.001, ^**^ indicates *P* < 0.01and ^*^ indicates *P* < 0.05

As seen in Table 4, univariate analysis of the general demographic and built environment showed statistically significant differences in gender, residential density, accessibility, pedestrian safety, aesthetics and security (*P* < 0.05), while diversity, traffic hazards, and obstruction were not statistically significant (*P* > 0.05). The above results of the univariate analysis suggest that gender, residential density, accessibility, pedestrian safety, aesthetics, and security will be considered in multinomial logistic regression.

**Table 4:** A univariate analysis of the built environment influencing the leisure-time MVPA of adolescent

Variable		**Walking**	Leisure-time** MPA**	Leisure-time** VPA**	χ^2^	P
Gender	boy	486(35.6%)	655(48%)	223(16.3%)	28.87^***^	< 0.05
	girl	560 (44.3%)	617 (48.8%)	87(6.9%)		
Residential density		298(187.5 ~ 494.5)	377(227 ~ 516.5)	378(230.8 ~ 520)	45.19^***^	< 0.05
Diversity		2.91(2.21 ~ 3.52)	2.83(2.3 ~ 3.39)	2.87(2.4 ~ 3.43)	3.62	0.164
Accessibility		3(2.5 ~ 3.5)	3(2.5 ~ 3.75)	3(2.5 ~ 3.75)	22.52^***^	< 0.05
Pedestrian facilities safety		3(2.33 ~ 3.33)	3(2.5 ~ 3.5)	3(2.5 ~ 3.5)	21.94^***^	< 0.05
Aesthetics		2.67(2 ~ 3.33)	3(2 ~ 3.67)	3(2.25 ~ 3.67)	17.89^***^	< 0.05
traffic hazards		2.33(1.67 ~ 3)	2.33(1.67 ~ 3)	2.33(1.67 ~ 3)	3.16	0.206
security		3(2.33 ~ 3.67)	3(2.67 ~ 4)	3(2.67 ~ 4)	25.09^***^	< 0.05
obstruction		2.25(1.5 ~ 2.75)	2.25(1.75 ~ 2.75)	2(1.5 ~ 2.75)	1.807	0.405

^***^ indicates *P* < 0.001 and ^*^ indicates *P* < 0.05

The results were shown in Table 5. Girls were used as the reference category. Gender was associated with the leisure-time MVPA among adolescents. Boys’ leisure-time MPA was 0.221 times more likely to engage in leisure-time MPA than girls. Boys’ leisure-time VPA was 0.581 times more likely to engage in leisure-time VPA than girls.

**Table 5:** Multinomial logistic regression of the built environment and leisure-time MVPA of adolescents [OR values ( OR values 95% CI)

Variable	Leisure-time MPA			Leisure-time VPA		
	Regression coefficient	*P*	OR(95%*CI*)	Regression coefficient	*P*	OR(95%*CI*)
1 = boy	0.221	0.005	1.247(1.07–1.45)^***^	0.581	0	1.788(1.44–2.22)^***^
2-girl	0			0		
Residential density	0.001	0	1.001(1.001–1.002) ^***^	0.001	0	1.001(1.001–1.002) ^***^
Accessibility	0.05	0.457	1.051(0.921–1.2)	0.031	0.735	1.032(0.86–1.239)
Pedestrian facilities safety	0.037	0.618	1.038(0.896–1.202)	-0.098	0.34	0.906(0.74–1.109)
Aesthetics	0.062	0.321	1.064(0.941–1.203)	0.171	0.049	1.187(1–1.409) ^*^
Security	0.123	0.041	1.131(1.005–1.273) ^*^	0.136	0.107	1.145(0.971–1.351)

Girl and walking was used as the reference category, ^***^ indicates *P* < 0.001 and ^*^ indicates *P* < 0.05

The results were shown in Table 5. When walking was used as the reference category, adolescents with favorable safety were 1.31 times more likely to engage in leisure-time MPA. This suggests that more security is associated with higher leisure-time MPA among adolescents. This suggests that as security around the built environment increases, there is an increase in the likelihood that young people will engage in leisure-time MPA. Residential density, accessibility, pedestrian safety, and aesthetics do not have a significant effect on leisure-time MPA.

Using walking as the reference category, adolescents with favorable aesthetics were 1.187 times more likely to engage in leisure-time MPA. This suggests that more aesthetics is associated with higher leisure-time VPA among adolescents. The more aesthetic, the higher the tendency of adolescents to engage in leisure-time VPA. Residential density, accessibility, pedestrian safety, and security do not have a significant effect on leisure-time VPA.

## Discussion

Researchers have recently begun collecting accurate data on walking, cycling, family and yard activities, and sedentary activities to understand the impact of the built environment on adolescent leisure-time physical activities [30]. It is also increasingly recognized that in order to improve the rate of leisure-time physical activity, non-traditional public health professions such as urban design, urban planning, and transportation must be incorporated into research and practice.

The results of this study showed that there were differences in leisure-time MVPA and perceptions of the built environment among adolescents of different genders. From the perspective of gender, boys engaged in leisure-time MVPA more than girls [31]. Our findings Consistent with previous studies in other Chinese cities [32], gender was positively associated with school-age children's physical activity in Shanghai [33]. More attention should be paid to the leisure-time MVPA of girls, to further stimulate them to participate in leisure-time MVPA. Transportation options and walkability of the neighborhoods, which require subjective judgment, were significantly associated with adolescent LTPA [34].

Security has been found to be an important factor in leisure-time MPA among adolescents. Security refers not only to the space with lighting, surveillance devices, traffic safety, and policing but also to the low risk of social crime. Greater security is associated with higher leisure-time MPA among adolescents. This research conclusion also applies to the elderly, the elderly living in communities with better security (0.110) have more leisure activities, so maintaining community security is a necessary condition to increase the duration of leisure activities for the elderly [35].Environments with high social security risks and criminal behavior are not suitable for adolescents to engage in physical activity [36]. Given that children are more active outdoors than indoors, Central European countries are committed to building communities with high levels of safety and walkability by creating safe public transportation and supporting safe active transportation for adolescents to get to school [37]. For children, an association has been demonstrated between cycling and the promotion of safe routes to school. With adolescents being significantly more sensitive to the security of the built environment, it is important to improve the traffic safety and security elements around the built environment. When adolescents can clearly perceive that the built environment is more secure, they will be more willing to engage in leisure-time MPA, and it will be easier to form partnerships for leisure-time MPA, effectively bringing them closer to each other psychologically and avoiding dangerous accidents such as violent crimes. It is also necessary to improve the public’s visibility of the built environment, increase the effectiveness of social control, and make every effort to maintain the safety of adolescents.

The results show that there is a close relationship between the aesthetically designed environment and the leisure-time VPA of adolescents.Aesthetics of the environment refers to residents' subjective perceptions of the natural surroundings of their homes, the beautification of public greenways; and the comfort level of their environment. Our findings are consistent with other research that found aesthetics is one of the important reasons for adolescents selecting leisure-time VPA [38, 39]. Another study, reported that people who enjoyed the scenery (observing interesting objects) while walking were more likely to engage in 150min or more of weekly LTPA [40]. This suggests that the neighborhood's high standard of aesthetics may have a favorable impact on the LTPA. Access to recreational amenities and aesthetics are both often acknowledged factors in leisure walking, and both are reliably linked to leisure-time VPA [41, 42]. Wright and colleagues found that having shaded footpaths, low traffic in the neighborhood, and being in an attractive area with street trees, wide grassy verges, and parks appeared to encourage walking. Many aesthetically, services and facilities around the community can enhance the attractiveness of the community and increase adolescents’ sense of identification with the built environment. Adolescents who live in such a built environment will change their awareness of participation in leisure-time VPA and their physical activity behavior. Studies have found that pleasant landscapes promote leisure walking as well as other forms of leisure-time VPA [43]. Within a walkable spatial context, being closer to a fountain, lake, beach, or dyke may result in increased walking time, and these features of the built environment can promote leisure-time VPA among adolescents. Streetscapes are an important aspect of environmental aesthetics, and distinctive human amenities, lighting design, cleanliness, architectural elements, and natural elements can all add to their attractiveness, with better streetscapes contributing to increased leisure-time VPA [44]. This study has shown that the aesthetics of the built environment can contribute to the enhancement of leisure-time VPA among adolescents, but that the built environment varies greatly across the vast territory of the country, with significant differences in the overall condition of road greenery, sanitation, lighting, and grading from city to city [45]. Therefore, it is important to improve the aesthetic quality of the built environment in urban communities.

Once the built environment meets the LTPA needs of adolescents, the marginal effects may weaken. Adolescents' preference for a sedentary lifestyle may not change their behavior, even if they live in a built environment suitable for engaging in LTPA. Alternatively, creating a built environment suitable for LTPA may result in more significant changes than we have found. Therefore, it is necessary to create a built environment suitable for adolescents to engage in LTPA, which may attract adolescents who like a sedentary lifestyle to engage in LTPA. The main directions for further research in this study are: Horizontal comparative research. Using Arc GIS technology, select different communities with spatial heterogeneity for cross-sectional comparison to explore the relationship between the built environment and the LTPA of adolescents. This perspective and method can be expanded and deepened in future research. In addition, research on longitudinal changes The next step in research can be a longitudinal study from a time perspective, studying the spatiotemporal changes in the type, quantity, spatial distribution, and other aspects of the built environment, and analyzing the impact of the built environment on the LTPA of adolescents.

The results of this study need to be interpreted in light of some limitations. First, the cross-sectional study design does not permit causal inferences. There have been studies that have proven longitudinal, higher park coverage was associated with smaller decreases in children's MVPA minutes per day [46, 47]. Secondly, although intentionally recognized questionnaires such as NEWS-CC and IPAQ were used in the present study, the self-reported measurement tools may introduce some measurement errors and self-report bias inevitably. Future research can use more objective measures of exposure and outcome variables. Third, demographic characteristics(race/ethnicity,age) and social psychological(income, anxiety and depressive symptoms) factors were not combined to explain the effect of the built environment on adolescents LTPA in this study[48]. Variables on demographics, socioeconomic status, etc. will be added in future studies. Fourth, the authors did not consider is the possibility that adolescent physical activity may occur in contexts other than the home, e.g., school, parks, etc. [49, 50]. The findings may also not be generalizable to other countries or more urban or regional areas.

## Conclusions

There was an association between the built environment and adolescents’ leisure-time MVPA. Potential association between residential density, accessibility, pedestrian safety, aesthetics, security, and adolescents' leisure-time MVPA. Security was associated with leisure-time MPA among adolescents. Aesthetics were associated with leisure-time VPA in adolescents. They both have a significant positive correlation.

My findings can provide useful insights for urban planners and public health professionals. For example, 1. Create safe shared use paths that separate walking, cycling, and motor traffic and provide adequate lighting and monitored paths to provide an additional sense of security. 2. Build bike paths and trails and design more walkable communities. 3. Develop safe routes to school to make the journey to and from school safer and more active. 4. Increasing green space and encouraging the design of public art installations improve the aesthetics of the community and provide opportunities for youth to participate in enjoyable and healthy sports activities. Urban planners and public health professionals can create more active, aesthetically pleasing communities that encourage adolescents to become more active in recreational physical activity, improving their overall health and well-being.

## Data Availability

The raw data supporting the conclusions of this article can be made available by the authors Ruiwang(wangrui_0529@163.com), without undue reservation.

## References

[1] Law M (2002). Participation in the occupations of everyday life. Am J Occup Ther.

[2] Strong WB, Malina RM, Blimkie C, Daniels SR, Trudeau F (2005). Evidence based physical activity for school-age youth. J Pediatr.

[3] Vuillemin A, Boini SP, Bertrais S, Tessier S, Oppert JM, Hercberg S, Guillemin F, Brianon S (2005). Leisure time physical activity and health-related quality of life. Prev Med.

[4] Iwasaki Y (2006). Counteracting stress through leisure coping: a prospective health study. Psychol Health Med.

[5] Bauman A, Allman-Farinelli M, Huxley R, James WPT (2010). Leisure-time physical activity alone may not be a sufficient public health approach to prevent obesity–a focus on China. Obes Rev.

[6] Petr B, Geckova AM, Sigmundova D, van Dijk JP, Reijneveld SA (2015). When children play, they feel better: organized activity participation and health in adolescents. BMC Public Health.

[7] Whitt-Glover MC, Taylor WC, Floyd MF, Yore MM, Yancey AK. Disparities in physical activity and sedentary behaviors among US children and adolescents: prevalence, correlates, and intervention implications. J Public Health Policy 2009, 2009,30, (-), S309-S334. 10.1057/jphp.2008.46 19190581

[8] Cui Z, Bauman A, Dibley MJ (2011). Temporal trends and correlates of passive commuting to and from school in children from 9 provinces in China. Prev Med.

[9] Marybeth G, Muldoon OT, Judith P (2015). An integrative review of social and occupational factors influencing health and wellbeing. Front Psychol.

[10] Sallis JE, Cervero RB, Ascher W, Henderson KA, Kraft MK, Kerr J (2006). An ecological approach to creating active living communities. Annu Rev Public Health.

[11] Salmon J, Timperio A (2007). Prevalence, trends and environmental influences on child and youth physical activity. Med Sport Sci.

[12] Zou Y, Ma Y, Wu Z, Liu Y, Xu M, Qiu G, Vos H, Jia P, Wang L (2021). Neighbourhood residential density and childhood obesity. Obes Rev.

[13] Dessing D, Pierik FH, Sterkenburg RP, van Dommelen P, Maas J, de Vries SI (2013). Schoolyard physical activity of 6–11 year old children assessed by GPS and accelerometry. The international journal of behavioral nutrition and physical activity.

[14] Sallis JF, Cervero RB, Ascher WW, Henderson KA (2006). An ecological approach to creating active living communities. Annu Rev Public Health.

[15] Frank LD, Sallis JF, Conway TL, Chapman JE, Saelens BE, Bachman W (2006). Many pathways from land use to health: associations between neighborhood walkability and active transportation, body mass index, and air quality. J Am Plann Assoc.

[16] Cervero R (1996). Mixed land-uses and commuting: evidence from the american housing survey. Transportation Research Part A: Policy and Practice.

[17] An RP, Shen J, Yang QY, Yang Y (2019). Impact of built environment on physical activity and obesity among children and adolescents in China: a narrative systematic review. J Sport Health Sci.

[18] Rodríguez DA, Cho GH, Evenson KR, Conway TL, Cohen D, Ghosh-Dastidar B, Pickrel JL, Veblen-Mortenson S, Lytle LA (2012). Out and about: association of the built environment with physical activity behaviors of adolescent females. Health Place.

[19] Da Silva ICM, Hino AA, Lopes A, Ekelund U, Brage S, Gonçalves H, Menezes AB, Reis RS, Hallal PC (2017). Built environment and physical activity: domain- and activity-specific associations among Brazilian adolescents. Bmc Public Health.

[20] Oyeyemi AL, Ishaku CM, Deforche B, Oyeyemi AY, Bourdeaudhuij ID, Dyck DV (2014). Perception of built environmental factors and physical activity among adolescents in Nigeria. Int J Behav Nutr Phys Act.

[21] Kelly P, Williamson C, Baker G, Davis A, Broadfield S, Coles A, Connell H, Logan G, Pell JP, Gray CM, Gill JM (2020). Cycle Nation Project. Beyond cycle lanes and large-scale infrastructure: a scoping review of initiatives that groups and organisations can implement to promote cycling for the Cycle Nation Project. Br J Sports Med.

[22] Laxer RE, Janssen I (2013). The proportion of youths' physical inactivity attributable to neighbourhood built environment features. Int J Health Geogr.

[23] Sandu P, Chereches RM, Baba CO, Revnic RN, Mocean F. Environmental influences on physical activity – Romanian youths' perspectives. Children and Youth Services Review 2018, 95.

[24] Shank JW (2010). Leisure as a context for active living, recovery, health and life quality for persons with mental illness in a global context. Health Promot Int.

[25] Mcgrath LJ, Hopkins WG, Hinckson EA (2015). Associations of objectively measured built-environment attributes with youth moderate-vigorous physical activity: a systematic review and meta-analysis. Sports Med.

[26] He G. Neighborhood built environment and physical activity in primary schoolchildren in Hong Kong. 2015.

[27] Brownson RC, Chang JJ, Eyler AA, Ainsworth BE, Kirtland KA, Saelens BE, Sallis JF (2004). Measuring the environment for friendliness toward physical activity: a comparison of the reliability of 3 questionnaires. Am J Public Health.

[28] Saelens BE, Sallis JF, Black JB, Chen D (2003). Neighborhood-based differences in physical activity: an environment scale evaluation. Am J Public Health.

[29] Cerin E, Sit CH, Cheung MC, Ho SY, Lee LC, Chan WM (2010). Reliable and valid NEWS for Chinese seniors: measuring perceived neighborhood attributes related to walking. Int J Behav Nutr Phys Act.

[30] Craig CL, Marshall AL, Sjöström M, Bauman AE, Booth ML, Ainsworth BE, Pratt M, Ekelund U, Yngve A, Sallis JF, Oja P (2003). International physical activity questionnaire: 12-country reliability and validity. Med Sci Sports Exerc.

[31] Wang K, Yang Y, Zhang T, Ouyang Y, Liu B, Luo J (2020). The Relationship Between Physical Activity and Emotional, ntelligence in College Students: The Mediating Role of Self-Efficacy. Front Psychol.

[32] Li M, Dibley MJ, Sibbritt D, Yan H (2007). Factors associated with adolescents' physical inactivity in Xi'an City, China. Med Sci Sports Exerc.

[33] Lin L (2018). Leisure-time physical activity, objective urban neighborhood built environment, and overweight and obesity of Chinese school-age children. J Transp Health.

[34] Huang X, Lu G, Yin J, Tan W. Non-linear associations between the built environment and the physical activity of children. Transportation Research Part D-Transport and Environment 2021, 98.

[35] Han H, Yang K, Yang C, Yang G, Xu L (2022). Influence and Mechanism of a Multi-Scale Built Environment on the Leisure Activities of the Elderly: Evidence from Hefei City in China. Int J Environ Res Public Health.

[36] Nicolas M, Oreskovic James M, Perrin Alyssa I, Robinson Joseph J, Locascio Jeff Blossom, Adolescents' use of the built environment for physical activity. BMC public health 2015, 15, 251. 10.1186/s12889-015-1596-6 PMC4369364 25880654

[37] Fraser S (2011). Cycling for transport and public health: a systematic review of the effect of the environment on cycling. Eur J Pub Health.

[38] Sugiyama T, Cerin E, Owen N, Oyeyemi AL, Conway TL, Dyck DV, Schipperijn J, Macfarlane DJ, Salvo D, Reis RS (2014). Perceived neighbourhood environmental attributes associated with adults recreational walking: IPEN Adult study in 12 countries. Health Place.

[39] Mendon?A G, Florindo AA, Rech CR, Freitas D. K. S. D, Farias Júnior J. C. d. Perceived neighborhood environmental characteristics and different types of physical activity among Brazilian adolescents. J Sports Sci 2017, 1–8. 10.1080/02640414.2017.1356024 28741451

[40] Cerin E, Saelens BE, Sallis JF, Frank LD (2006). Neighborhood environment walkability scale: validity and development of a short form. Med Sci Sports Exerc.

[41] B.H.M.S.c, M. D.; Mummery, K., Psychosocial and environmental factors associated with physical activity among city dwellers in regional Queensland. Preventive Medicine 2005, 40, (4), 363–372. 10.1016/j.ypmed.2004.06.017 15530589

[42] Gebel K, Bauman AE, Petticrew M (2007). The physical environment and physical activity: a critical appraisal of review articles. Am J Prev Med.

[43] Jiang YP, Sun HH (2021). Exploring the Characteristics and Influencing Factors of Leisure Walking Based on the Demand of Behavior. Sustainability.

[44] Buehler R (2012). Determinants of bicycle commuting in the Washington, DC region: the role of bicycle parking, cyclist showers, and free car parking at work. Transp Res Part D.

[45] Bird, E. L.; Ige, J. O.; Pilkington, P.; Pinto, A.; Petrokofsky, C.; Burgess-Allen, J., Built and natural environment planning principles for promoting health: an umbrella review. Bmc Public Health 2018, 18, (1), 930. 10.1186/s12889-018-5870-2 PMC6064105 30055594

[46] Yi L, Mason TB, Yang CH, Chu D, Dunton GF (2021). Longitudinal associations between neighborhood park and open space access and children's accelerometer-assessed measured physical activity: the evidence from the MATCH Study. J Phys Act Health.

[47] Buck C, Eiben G, Lauria F, Konstabel K, Page A, Ahrens W, Pigeot I (2019). Urban Moveability and physical activity in children: longitudinal results from the IDEFICS and I. Family cohort. Int J Behav Nutr Phys Act.

[48] Naya CH, Yi L, Chu D, Dunton GF, Mason TB (2022). Cross-sectional and longitudinal associations of park coverage, greenness exposure and neighbourhood median household income with children's depressive and anxiety symptoms. J Paediatr Child Health.

[49] Yi L, Wilson JP, Mason TB, Habre R, Wang S, Dunton GF (2019). Methodologies for assessing contextual exposure to the built environment in physical activity studies: a systematic review. Health Place.

[50] Smith L, Foley L, Panter J (2019). Activity spaces in studies of the environment and physical activity: a review and synthesis of implications for causality. Health Place.

